# Comparative analysis of clinical decision tools for acute pulmonary embolism at a tertiary care center

**DOI:** 10.6026/973206300220754

**Published:** 2026-02-28

**Authors:** Ashis Tiwari, Sukh Dayal Kumhar, Praveen Kumar Tagore, Jitendra Kanjolia, Shivani Parihar, Abha Bardiya

**Affiliations:** 1 Department of General Medicine, Government Medical College, Datia, Madhya Pradesh, India; 2 Department Of Pathology, Chirayu Medical College, Bhopal, Madhya Pradesh, India; 3 Department of Anaesthesiology, Shyam Shah Medical College, Rewa, Madhya Pradesh, India

**Keywords:** Pulmonary embolism, risk stratification, PESI, sPESI, mortality prediction, clinical decision rules

## Abstract

Acute pulmonary embolism (PE) requires accurate risk stratification to guide intensive care versus safe discharge decisions. Therefore, it is of interest to compare original Pneumonia Severity Index (PESI) versus Simplified Pneumonia Severity Index (sPESI) in 410 acute PE patients for predicting 30-day mortality and adverse outcomes. Both indices showed excellent negative predictive values (NPVs) (>98%) for mortality, with sPESI demonstrating higher sensitivity (94.1% vs 89.5%). Original PESI had slightly superior risk discrimination (AUC 0.78 vs 0.75), but sPESI proved more clinically practical. sPESI advances PE management as the preferred bedside tool for identifying low-risk patients suitable for outpatient care.

## Background:

The global prevalence of acute pulmonary embolism (PE) has grown in recent years and it now ranks third among acute cardiovascular syndromes, behind only myocardial infarction and stroke. Although there have been improvements in the diagnostic imaging and anticoagulant therapy, the acute PE has a high mortality rate within a period of time with estimated short term mortality being between 2 and more than 30 percent of those who present with cardiogenic shock [1]. PE has a very heterogeneous clinical presentation and the strong risk stratification approaches are required to make evidence-based site-of-care decisions. The main clinical issue is not only in the diagnosis but in the severity of the event to be able to allocate resources adequately; in particular, distinguishing low-risk patients that can be treated as outpatients and the high risk patients that require thrombolysis or intensive care unit (ICU) hospitalization [2]. In the past, in the assessment of severity, anatomical burden (e.g., clot load on CT angiography) was of great importance, but it is now established that clinical status and functional reserve have gained importance. In addressing this, some clinical decision rules (CDRs) are developed. Pulmonary Embolism Severity Index (PESI) created by Aujesky *et al.* is among the most thoroughly validated prognostic tools [3]. The PESI consists of 11 variables, the scores of which are weighted and it divides patients into five groups of risks (I-V). Although well proven, it is not always applicable in overcrowded emergency departments (ED) due to its complexity and the calculating nature of the method [4]. As a result, the Simplified PESI (sPESI) was created, cutting the criteria off to six variables that are equally weighted with an aim to be used at the bedside [5].

The latest recommendations such as the ones issued by the European Society of Cardiology (ESC) advise such scores to be used in managing patients [6]. There is however, research gap on comparative performance of these tools in real world tertiary care centres where patient groups have high burden of comorbidity including active malignancy or severe heart failure whose scoring systems may be different between validation cohorts [7]. Moreover, although the negative predictive value of these scores is well-established, their discriminatory proficiency in the intermediate-risk group which is a group frequently subject to fluctuate management tactics has been the focus of research [8]. Recent research has indicated that sPESI is not inferior to PESI in predicting mortality, but one of the limitations is that it can over-identify risk in older people, resulting in unnecessary hospitalization [9]. On the other hand, the complexity of the original PESI makes it possible to stratify more finely but requires full data sets which are not always accessible at the first stage of triage [10]. Therefore, it is of interest to provide a comparative study of the original PESI and the sPESI in the evaluation of the severity of proven acute PE in a tertiary care unit. In particular, we are going to compare their diagnostic accuracy in making predictions about 30-day all-cause mortality and composite adverse outcomes, which will indicate to us in what tool it is possible to achieve the best balance between accuracy and clinical utility.

## Materials and Methods:

A total of 410 consecutive adult patients were enrolled from Government medical College Datia M. P. India, Start date of the research was August 2025 and end date was October 2025. The sample size was calculated based on an estimated 30-day mortality rate of 10%, requiring a minimum of 380 patients to detect a 5% difference in sensitivity between the two scores with 80% power and a 5% alpha error.

## Inclusion criteria:

[1] Adults aged ≥ 18 years.

[2] Acute pulmonary embolism confirmed objectively with computed tomography. A high-probability Ventilation/Perfusion (V/Q) scan or a CTPA of the pulmonary artery tree which reveals a filling deficiency.

[3] Diagnosis confirmed within 24 hours of admission.

## Exclusion criteria:

[1] Patients receiving anticoagulation for PE for >48 hours prior to transfer.

[2] Incidental PE findings in asymptomatic patients.

[3] Pregnancy.

[4] Life expectancy < 3 months due to terminal illness (unrelated to PE) to avoid skewing mortality data.

[5] Incomplete clinical data preventing score calculation.

## Data collection and tools:

Baseline demographic data, vital signs, comorbidities and laboratory values were recorded at the time of diagnosis.

## The pulmonary embolism severity index (PESI):

Patients were stratified into five classes based on age, sex, cancer history, heart failure, lung disease, pulse >110/min, systolic BP <100 mmHg, respiratory rate >30/min, temperature <36°C, altered mental status and arterial oxyhemoglobin saturation <90%.

Total points determined the class:

[1] Class I (65 points) and Class II (66-85 points): Low Risk.

[2] Class III (86-105 points): Moderate Risk.

[3] Class IV (106-125 points) and Class V (>125 points): High Risk.

## The simplified PESI (sPESI):

Age >80 years, History of cancer, Chronic cardiopulmonary disease, Pulse >110/min, Systolic BP <100 mmHg and Arterial oxyhemoglobin saturation <90%.

[1] 0 points: Low Risk.

[2] ≥ 1 point: High Risk.

## Outcome measures:

Mortality from any cause at 30 days was the main goal. The secondary objective was a combination of unfavorable events within 30 days, which were defined as thrombolysis, recurrent PE, surgical embolectomy, mechanical breathing, or vasopressor support. On the 30th day, clinical data and telephone interviews were used for follow-up.

## Statistical analysis:

The statistical package SPSS, version 26.0 (IBM Corp, Armonk, NY), was used for the data analysis. The Student's t-test was used to compare continuous variables, which were reported as mean ± standard deviation (SD). The Chi-square test was used to assess categorical variables, which were given as frequencies or percentages. Receiver Operating Characteristic (ROC) curve analysis was used to examine the prognostic performance of PESI and sPESI. One way to compare discriminatory power was by calculating the Area under the Curve (AUC). The prediction of mortality was evaluated using the PPV, NPV, Sensitivity and Specificity metrics. It was deemed statistically significant if the p-value was less than 0.05.

## Results:

In the end, 410 individuals were considered who had acute PE verified. There was a little female preponderance (52.4%) and the average age of the group was 64.2 ± 15.8 years. Out of a total of 35 participants, 8.5% died within 30 days and out of 60, 14.6% had some kind of unfavorable result. The two most prevalent co-occurring conditions were active cancer (18.5%) and systemic hypertension (42.1%). The baseline characteristics of survivors and non-survivors are summarized in (Table 1). Non-survivors had a greater frequency of disturbed mental state, hypotension and tachycardia upon presentation, as well as being noticeably older (p < 0.001). Individuals were categorized based on their PESI and sPESI results. The original PESI included a Low Risk classification for 38.5% of patients (Classes I & II). Only 30.2% were deemed Low Risk (0 points) according to the sPESI. According to the results, more people were deemed High Risk by sPESI than by the original PESI. The mortality rates rose steadily as the risk groups became more severe. Class V had a mortality rate of 28.4 percent, in contrast to 0 percent in PESI Class I. The low-risk group had a mortality rate of 1.6% for sPESI, whereas the high-risk group had a rate of 11.5% (Table 2). The prognostic performance for predicting 30-day mortality is detailed in Table 3. The sPESI demonstrated a higher sensitivity (94.3%) compared to the original PESI (using Class III-V as positive cutoff: 89.5%), suggesting sPESI is safer for ruling out mortality risk. However, the PESI showed higher specificity (42.4% versus 32.8%). Compared to sPESI's 0.75 (95% CI: 0.68-0.82) AUC, PESI's 0.78 (95% CI: 0.71-0.85) was marginally higher. In terms of overall discrimination, PESI had a numerical advantage, but when comparing pairs, the difference was not statistically significant (p = 0.12). With NPVs > 98%, both ratings were very promising.

## Discussion:

The present prospective study results in a tertiary care unit support the value of the original PESI as well as the Simplified PESI (sPESI) as efficient prognostic instruments in the acute pulmonary embolism. According to our comparative analysis, the two scores have a great negative predictive value (NPV), which qualifies them as a solid measure of identifying low-risk patients, but they are slightly different in specificity and in the distribution of risks. The major advantage of the sPESI as reflected in our findings is its high sensitivity (94.3) and easy usage. The sPESI will help ease the computational load on clinicians by narrowing down its criteria to six variables. Our results can be compared to a validation study which states that ranked sPESI as an alternative that is non-inferior to original PESI in the process of eliminating adverse outcomes [5]. This high NPV (98.4) shows that a patient with a sPESI of 0 faces a low risk of death, which explains why the practices of early discharge or outpatient management are not dangerous to this particular group. It is clinically relevant in resources constrained environments where there is limited availability of ICU beds [11]. Nonetheless, the lack of complexity of sPESI is connected to low specificity (32.8% vs 42.4% of PESI). Our cohort was almost 70% High Risk (≥ 1 point) by the sPESI. This dichotomous (Low versus High) classification is not as fine as the five classes of the original PESI. A patient suspected of having a single risk factor (e.g. age 81) is classified with a patient in cardiogenic shock, both having a "High Risk" sPESI designation. Such an over-triage phenomenon has been observed earlier in the literature, indicating that sPESI is very useful at the exclusion level but not at determining the level of risk among the positive cohort [12]. With the five strata in the original PESI, the intermediate- risk patients (Class III) and very high-risk patients (Class V) can be better distinguished, which can be used to determine whether to provide the patient with ward admission or ICU care [13]. In our study, we have found slightly better AUC with the original PESI (0.78) than with sPESI (0.75). Weighted variables added into the original PESI gives a better portrayal of the cumulative physiological burden [14]. This could be because signs of systemic hypoperfusion and sepsis, which sPESI might fail to detect in complex patients and that may be present due to altered mental status and temperature in the original PESI are included in the original PESI [15]. Additional context for these scores should be provided by the European Society of Cardiology's (ESC) proposed modern risk stratification system. Imaging (RV dysfunction) and biomarkers (Troponin/BNP) should be used in conjunction with these clinical ratings, according to the ESC recommendations [16]. Despite the fact that we just looked at clinical ratings in our investigation, new evidence suggests that patients classified as intermediate-risk by PESI Class III/IV should undergo further testing for biomarkers of subclinical myocardial damage [17]. Higher troponin levels have a significant impact on enhancing future prognosis of sPESI, forming a high-intermediate-risk category that should be given more attention [18]. Weaknesses of our research are that it is a single-center observational study. The tertiary care environment must have caused a referral bias, which means that the cohort was more burdened with comorbidities (cancer, heart failure) than a general community hospital, which might have increased the percentage of high-risk patients. Also, we failed to examine the effects of the individual treatments (e.g., thrombolysis versus anticoagulation alone) on predicting accuracy of the scores [6, 19]. Lastly, the research was not adequately powered to identify the differences in non-fatal recurrence rates, but these were incorporated in the composite outcome.

## Conclusion:

Due to its high sensitivity and excellent negative predictive value, the sPESI is better suited for use in emergency practice. But since it is binary, it puts a high percentage of patients at high risk. Although complex, the original PESI provides a better specificity and discriminative ability, which is more appropriate in grading the severity of the admitted patients in order to determine the intensity of monitoring in inpatients.

## Figures and Tables

**Table 1:** Baseline clinical characteristics of study population (N=410)

**Variable**	**Total (N=410)**	**Survivors (N=375)**	**Non-Survivors (N=35)**	**P-value**
Age (years), Mean ± SD	64.2 ± 15.8	62.1 ± 14.5	76.8 ± 11.2	<0.001
Male Sex, n (%)	195 (47.6)	178 (47.5)	17 (48.6)	0.91
Active Cancer, n (%)	76 (18.5)	60 (16.0)	16 (45.7)	<0.001
Heart Failure, n (%)	65 (15.9)	55 (14.7)	10 (28.6)	0.03
Systolic BP < 100 mmHg, n (%)	45 (11.0)	25 (6.7)	20 (57.1)	<0.001
Heart Rate ≥ 110 bpm, n (%)	92 (22.4)	72 (19.2)	20 (57.1)	<0.001
SaO2 < 90%, n (%)	88 (21.5)	70 (18.7)	18 (51.4)	<0.001

**Table 2:** Mortality rates according to PESI and sPESI risk stratification

**Scoring System**	**Risk Category**	**N (%)**	**Mortality n (%)**	**P-value (Trend)**
PESI				<0.001
	Class I (Very Low)	65 (15.9)	0 (0.0)	
	Class II (Low)	93 (22.7)	2 (2.1)	
	Class III (Intermediate)	98 (23.9)	6 (6.1)	
	Class IV (High)	80 (19.5)	8 (10.0)	
	Class V (Very High)	74 (18.0)	19 (25.7)	
sPESI				<0.001
	Low Risk (0 points)	124 (30.2)	2 (1.6)	
	High Risk (≥ 1 point)	286 (69.8)	33 (11.5)	

**Table 3:** Diagnostic performance for predicting 30-day mortality

**Statistic**	**PESI (Cut-off Class ≥ III)**	**sPESI (Cut-off Score ≥ 1)**
Sensitivity (%)	94.3	94.3
Specificity (%)	42.4	32.8
Positive Predictive Value (PPV) (%)	13.1	11.5
Negative Predictive Value (NPV) (%)	98.7	98.4
Accuracy (%)	46.8	38
AUC (95% CI)	0.78 (0.71-0.85)	0.75 (0.68-0.82)
Note: For PESI
sensitivity calculation,
Classes III, IV and V
were considered "High Risk"
for adverse outcome potential
